# Using Implementation Mapping to increase uptake and use of *Salud en Mis Manos*: A breast and cervical cancer screening and HPV vaccination intervention for Latinas

**DOI:** 10.3389/fpubh.2023.966553

**Published:** 2023-03-15

**Authors:** Lara S. Savas, Preena Loomba, Ross Shegog, Angelita Alaniz, Crystal Costa, Emily Adlparvar, Marlyn A. Allicock, Roshanda Chenier, Margaret Goetz, Christine M. Markham, Maria E. Fernandez

**Affiliations:** ^1^Department of Health Promotion and Behavioral Sciences, University of Texas Health Science Center at Houston (UTHealth) School of Public Health, Houston, TX, United States; ^2^Center for Health Promotion and Prevention Research, UTHealth School of Public Health, Houston, TX, United States; ^3^Department of Health Promotion and Behavioral Sciences, UTHealth School of Public Health, Dallas, TX, United States; ^4^ProSalud, Inc., Houston, TX, United States

**Keywords:** Implementation Mapping, implementation strategy, health equity, community health workers, cancer prevention, cervical screening, mammography, HPV vaccination

## Abstract

**Background:**

Despite CDC recommendations for breast and cervical cancer screening and HPV vaccination, cancer control behaviors are underutilized among low-income Latinas. *Salud en Mis Manos* (SEMM), adapted from *Cultivando La Salud*, is a community health worker- (CHW-) delivered evidence-based intervention (EBI), shown to increase breast and cervical cancer screening.

**Methods:**

We used Implementation Mapping to create SEMM-Dissemination and Implementation Assistance (SEMM-DIA), a set of implementation strategies designed to support implementation and maintenance of SEMM in clinic settings. Specifically, we used Implementation Mapping's five iterative tasks to guide the use of theories and frameworks, evidence, new data, and stakeholder input to develop strategies to accelerate and improve implementation fidelity, reach, and maintenance of the SEMM intervention. The resulting implementation mapping logic model also guides the SEMM-DIA evaluation plan to assess reach, effectiveness, implementation, and maintenance.

**Discussion:**

Increased use of implementation planning frameworks is necessary to accelerate the translation of EBIs to public health practice. This work demonstrates the application of Implementation Mapping to develop SEMM-DIA, providing a model for the development of other implementation strategies to support translation of evidence-based health promotion interventions into clinic settings.

## Introduction

Despite the availability and effectiveness of evidence-based interventions (EBIs), their implementation and dissemination have been slow, resulting in limited reach (1), and missed opportunities for positive public health impact (2–4). Challenges to EBI adoption, implementation, and maintenance are multifactorial and multilevel, and are influenced by environmental and organization-level factors (e.g., resources and capacity), as well as individual implementer-level factors (e.g., skills or self-efficacy). Implementation support strategies designed to address the complex factors that influence EBI adoption, implementation, and maintenance can promote translation of behavioral intervention research to effective public health practice.

Implementation strategies provide guidance and support to EBI adopters and implementers, helping to ensure effective program delivery, including attention to fidelity, such that essential elements of the intervention are preserved as they are implemented within their organization's context. Implementation strategies must also build on organizations' assets and address organizations' needs (2–4). We used Implementation Mapping, a framework for planning and developing implementation strategies to accelerate and improve implementation and maintenance of *Salud en Mis Manos* (SEMM), an evidence-based community health worker (CHW)-delivered intervention shown to increase breast and cervical cancer screening among low-income Latinas (5, 6). The Implementation Mapping framework guides a systematic planning process that incorporates perspectives and experiences of multiple stakeholders and uses evidence and theory to inform development of implementation strategies (7). While the SEMM intervention addresses an important problem (underutilization of breast and cervical cancer screening) and has the potential to reduce breast cancer survival disparities and the disproportionate burden of cervical cancers among Latinas (compared with non-Hispanic whites; NHWs) (8), widespread implementation of SEMM has been slow.

Briefly, SEMM is an evidence-based intervention based on *Cultivando la Salud* (CLS), a CHW-delivered breast and cervical cancer screening behavioral intervention originally developed for Mexican-American women living in farmworkers communities (9, 10). Adaptations of SEMM for medically underserved Latinas in urban and suburban settings increased the behavioral intervention's generalizability to Latinas from diverse backgrounds and to those living in areas with different environmental and social contexts (6). SEMM intervention planners adapted the original CLS CHW-delivered education intervention and referral protocol (to deliver referrals to low-cost services) guided by the Intervention Mapping framework for adaptation (IM ADAPT). This systematic approach to intervention adaptation planning informed integration of theory, evidence, and formative work to ensure retention of salient elements while increasing relevance to the new population and setting. In addition, the SEMM adaptation included development of a telephone-based health coaching and navigation component delivered by health coach navigators trained to help women overcome structural and personal barriers to completing needed cancer prevention services. Based on a randomized controlled trial (Cancer Prevention and Research Institute of Texas, CPRIT award, PP110081), the adapted intervention effectively increased screening in the intervention compared with control groups for both mammogram (39.9 vs. 20.3%; *p* < 0.001) and Pap outcomes (55.8 vs. 27.4%; *p* < 0.001); intent-to-treat analyses were also significant (11). While proven effective, broad uptake and use of SEMM has been slow and implementation in clinical settings has been particularly limited.

We used Implementation Mapping, a systematic process for designing and tailoring implementation strategies to develop *Salud en Mis Manos-* **D**issemination and **I**mplementation **A**ssistance (SEMM-DIA), a multifaceted implementation strategy, to support implementation of SEMM. This paper serves as a model for applying the Implementation Mapping framework to develop implementation strategies. In the case of SEMM-DIA, these strategies were designed to build capacity of clinic leadership and management, intervention champions, and CHWs to plan, manage, implement, and maintain SEMM.

## Methods

### Conceptual framework and theoretical basis for the development of the implementation strategy

**Figure 1:**
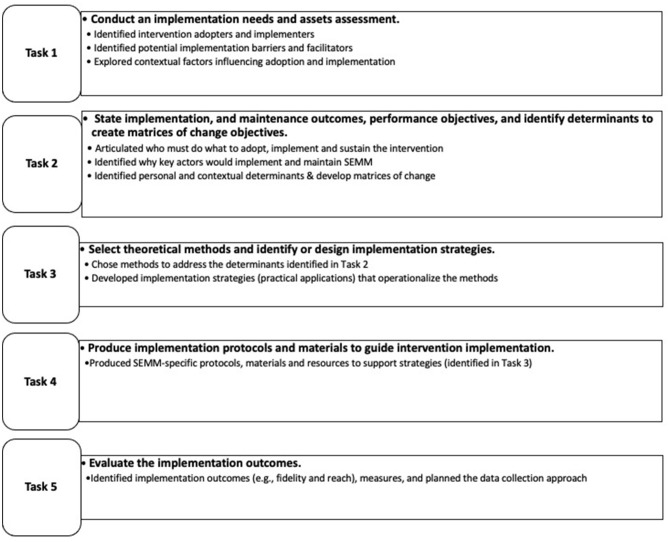
Implementation Mapping tasks and key deliverables.

The Implementation Mapping framework includes five tasks that guide implementation strategy planners in the design and tailoring of implementation strategies. These tasks are described below (see Figure 1) (7). Implementation Mapping is a step-by-step protocol that incorporates empirical evidence, stakeholder input and feedback, and is informed by theories, models and frameworks. In the development of SEMM-DIA, we used the Implementation Mapping framework to help integrate behavioral theory [i.e., Social Cognitive Theory (SCT)], to identify behavioral determinants at multiple levels (e.g., organization and CHW) (12) and implementation frameworks, including the Interactive Systems Framework for Dissemination and Implementation (ISF) (13) and the Exploration, Preparation, Implementation, and Sustainment (EPIS) framework (14). To guide planning evaluation outcomes, we used RE-AIM, focusing on Reach, Effectiveness, Implementation, and Maintenance (intention) (1). All behavioral and Implementation Science theories and frameworks used to develop the SEMM-DIA implementation support strategy are summarized in Table 1.

**Table 1:** Summary of theories, frameworks and models used to guide development of SEMM-DIA, an implementation intervention.

**Task #**	**Implementation science theories and frameworks**	**Role in informing implementation strategy design and/or evaluation planning**
Task 1–5	Social Cognitive Theory (SCT) (15)	- Helps to identify individual-level behavioral determinants (e.g., motivation, self-efficacy, and behavioral capability) at multiple levels (e.g., individual, and organizational levels) - Identifies corresponding methods for influencing determinants to change behavior (e.g., modeling verbal persuasion, and skills training)
Task 2	Interactive Systems Framework (ISF) (13, 16)	- Explains the process of introducing and implementing a health intervention into a new practice setting by describing three systems and processes required to support dissemination and implementation: (1) the synthesis and translation system; (2) the prevention support system; and (3) the delivery system (e.g., the clinics with CHWs)
Task 2 and 4	Exploration, Preparation, Implementation, and Sustainment (EPIS) framework (14)	- Guides the implementation process and identifies levels within and across organizational contexts - Provides a basis for ordering IM program performance objectives (who must do what to implement SEMM; specified in Task 2) - Guides and describes the SEMM implementation process (specified in Task 4)
Task 5	Reach, Effectiveness, Implementation, and Maintenance (RE-AIM) framework	- Guides planning of the evaluation, including reach, effectiveness, level of adoption and implementation outcomes

The Implementation Mapping planning process also supports a community-engaged approach to implementation strategy development, helping to integrate multiple stakeholder perspectives throughout development. Using a community-engagement approach, we included perspectives of stakeholders with previous experience implementing the SEMM intervention, as well as people with insight into the clinic practice setting (e.g., clinic leaders, clinic managers, and CHWs) who could also represent the patient perspective. Implementation stakeholders invited to participate in the planning process included managers working with The Breast and Cervical Cancer Collaborative of Texas, and representatives from the Centers for Disease Control and Prevention (CDC)-funded Texas Prevention Research Center Community Advisory Board (CAB). The CAB included community leaders representing community-based CHW organizations (e.g., ProSalud, Inc. and South Coastal Area Health Education Center; AHEC), and CHWs with substantial field experience working with Latinas on breast and cervical cancer screening interventions in Federally Qualified Health Centers (FQHCs) in the Greater Houston area. It also included staff and leaders at community health centers, many of whom provided insights into the patient populations they serve, such as patient's concerns regarding undergoing cancer screening and barriers to completing screening services.

### Methods for each Implementation Mapping task

#### Task 1. Conduct an implementation needs and assets assessment

The research team conducted 12 semi-structured Zoom-based interviews with clinic personnel representing clinic leadership (e.g., CEO, Medical Director), mid-management (e.g., Clinic Program Manager, MA/Director of Program Development, and CHWs at four different Texas Community Health Centers. Participants were provided with a five-minute PowerPoint overview of the SEMM intervention prior to their interviews. Three interview guides were developed respectively for leadership, mid-level, and CHWs, informed by SCT, ISF and the Readiness heuristic, R = MC^2^ (readiness = motivation × innovation specific capacity × general capacity) (15). Interview questions focused on exploring clinic and program implementers' needs and assets (e.g., resources, infrastructure, and potential related experiences) that may influence SEMM implementation, such as: (1) What could be potential problems/barriers that you might face to implement the intervention? (2) Who would be involved in planning how the program would be incorporated into clinical workflows and practice? (3) What would make it easier to adopt and implement the intervention? and (4) What makes an organization ready (to take on a program like this/new programs)? Interviews were audio-recorded and professionally transcribed. Using an adapted rapid qualitative approach (RQA) (16), one independent reviewer (reviewer CC) analyzed all 12 interview transcriptions to identify potential implementation barriers and facilitators. Transcript data were tabulated in summary tables by content domain. For example, the question, “Your clinic has CHWs—can you tell me a bit about the program and how CHWs are used?” represents the domain “CHW utilization.” The data were then coded for potential barriers and facilitators. A second reviewer (reviewer PL) reviewed the summary tables for clarity. Both reviewers then met to discuss and reach consensus on any discrepancies. The data were stratified by implementer level (leadership, mid-level, CHW) and by theoretical constructs (e.g., complexity and staff capacity (Readiness constructs). To further examine the data, an in-depth content and thematic analysis is currently being conducted by the research team using a traditional qualitative analysis approach. Findings will help better understand which additional environmental factors should be considered for improved program implementation and maintenance (17–20).

We also used core processes adapted from Intervention Mapping to aid in the identification of barriers and facilitators to implementation (21). As described by Fernandez et al., “Core processes are a set of helpful actions or tools that can provide a systematic way to answer questions raised during the planning process and aid in the identification of potential barriers and facilitators to implementation” (21, 22). These core processes were used throughout the five tasks of implementation mapping where appropriate and were fundamental in Task 1. They included: (1) Brainstorm potential factors (i.e., barriers and facilitators) based on experience, past needs assessments, and published literature, (2) Use theories and frameworks, (3) Collect new data, and (4) Prioritize the most important and changeable factors. We considered both health behavior theory (i.e., SCT) and implementation science frameworks (i.e., Interactive Systems Framework and Organizational Readiness) during the identification of factors potentially influencing implementation. Doing so enabled the research team to confirm if the listed barriers and facilitators previously identified aligned with constructs from existing theoretical models. This step also informed the identification of additional constructs that were relevant in similar implementation efforts and allowed the research team to refine performance objectives (who must do what to implement SEMM). Finally, the research team integrated diverse perspectives provided by the CAB members.

#### Task 2. State implementation and maintenance outcomes, performance objectives, and identify determinants to create matrices of change objectives

In Task 2, based on the needs and assets assessment conducted in Task 1, the research team articulated performance objectives (who must do what to implement SEMM) as well as the potential factors (from theory, evidence, and new data) that might influence key actors' pre-implementation, implementation, and maintenance of SEMM. We developed matrices of specific change objectives by crossing performance objectives and determinants and asking, “what has to change in X determinant in order to accomplish this implementation performance objective.”

#### Task 3. Choose theoretical methods; and identify or design implementation strategies

In Task 3, we considered theoretical change methods (both those focused on changing behavior and those focused on influencing the implementation environment) that could address determinants identified in Task 2 (23, 24). We developed the implementation strategies by operationalizing the methods. We created practical applications of those methods such that they were feasible and appropriate for use in clinic settings. This task was also conducted in partnership with stakeholder engagement, e.g., those with previous experience implementing SEMM, as well as clinic and CHW experiences in general. The research team engaged CAB members monthly *via* Zoom to pose a series of questions/ideas/implementation strategies to CAB members to gain their insight into what resources would best serve and support clinic personnel with the implementation of SEMM. By working with clinic representatives, selection of implementation strategies took into consideration relevance and feasibility for different implementers in clinical settings.

#### Task 4. Produce implementation protocols and materials to guide intervention implementation

Following the planning of implementation strategies in Task 3, we identified, adapted, or produced the SEMM implementation protocols, materials, and tools to include in the SEMM-DIA implementation package. This task was also informed by the EPIS “meta” framework (14). This “meta” framework consists of five phases that we used to order SEMM-DIA performance objectives, including (1) Exploration (Prioritizing SEMM), (2) Preparation (Assessing clinic readiness), (3) Preparation for implementation of SEMM, (4) Implementation, and (5) Maintenance. Each phase was associated with clinic personnel responsible for that phase (i.e., clinic leadership, SEMM program manager and/or champion, and CHW). We also developed documentation to support clinic stakeholders' implementation planning and process monitoring of SEMM. The overarching goal of this implementation strategy package was to provide clear, user-friendly support to promote feasibility, and fidelity of implementation.

#### Task 5. Evaluate the implementation outcomes

Task 5 of Implementation Mapping focused on planning the evaluation of the SEMM-DIA implementation strategy, to assess the effect of SEMM-DIA on implementation outcomes, and on SEMM effectiveness outcomes (e.g., breast and cervical cancer screening and HPV vaccination). We also developed indicators and measures for the evaluation, informed by the matrices. Our evaluation plan included measures to assess organizational readiness for implementation, level of implementation, determinants of implementation, experiences with implementing SEMM, and implementation maintenance. Selection of mediators and moderators of implementation was guided by behavioral theoretical constructs based on SCT and ISF identified during the planning process.

## Results

### Task 1. Conduct needs and asset assessment

Stakeholder engagement played a critical role on the planning team (comprised of both stakeholders and research team members). The CAB weighed in on key actionable findings to ultimately inform implementation strategy development. Input from all CAB members during Zoom meetings helped to identify potential barriers and facilitators influencing the implementation and maintenance of the program. Included in these CAB meetings over the course of the needs assessment period were clinic leadership, clinic managers, as well as former SEMM CHW managers (heretofore referred to as SEMM champions).

#### Adopters and implementers

Potential barriers and facilitators to implementation corresponded to Readiness and SCT constructs. For example, related to Readiness, staff capacity, and complexity constructs were identified as potential barriers. Participants expressed concerns about staff capacity and the need to further expand CHWs' role for program implementation and having to hire new clinic staff, “What we need are new people to perform this role, I don't have people I could add more responsibility to.” Another potential barrier included complexity, as it is related to data management. Participants had concerns about data risk management and data protection (e.g., who will be responsible for acquiring and securing the program's database?).

#### Facilitators

Regarding potential facilitators, leadership participants stressed the importance of intra-organizational relationships, stating that obtaining clinic staff buy-in for intervention implementation is important, “I'd also gain the feedback from people who will implement it, so that we can be on the same page that we're going to do it.” Other potential facilitators related to SCT included positive attitudes among participants who recognized that having CHWs is instrumental, “Our community health workers are used in every capacity of the organization, from our clinic services, health education, outreach, they are the ones who are instrumental in doing the education and outreach activities for the clinics.” Participants emphasized CHWs' role as one that can “wear multiple hats” and therefore would likely be able to play various roles related to implementation. Positive attitudes also included the belief that having a SEMM champion is critical for its success. Of note, participants also discussed the need to develop communication strategies to facilitate SEMM intervention promotion and implementation by clinic staff, “This is what I can just easily send [referring to email templates] to the staff. This is what we're doing and how to refer a patient kind of things.”

#### Barriers

The planning team, including researchers and CAB stakeholders, (e.g., clinic staff, SEMM champions, and CHWs) prioritized which barriers needed to be addressed. Clinic participants provided insight into addressing implementation challenges and shared lessons learned and practical suggestions regarding factors affecting CHW implementation. For example, in one of the monthly CAB meetings, stakeholders validated the finding that CHWs do, in most cases, “wear multiple hats.” Stakeholders also added that when there is no CHW, they often have other staff (e.g., patient navigator, patient educators) who could (and do) serve in a similar role. While the original program was designed to focus on community outreach for identifying women in need of services, CAB members stressed the importance of in-reach (i.e., focusing on current clinic patients), in addition to outreach as an important way to identify women in need of screening and HPV vaccination.

CAB members also helped clarify who the potential implementers in clinic practice settings would likely be in the safety-net clinic context (e.g., FQHCs). CHW managers with extensive experience managing CHW training and CHW delivery provided insight into potential barriers and facilitators to managing CHWs. CAB members discussed the importance of SEMM champions engaging in weekly meetings with CHWs, in which they use effective facilitation skills, such as facilitating discussions between CHWs to encourage CHWs to share their work challenges and successes. For clinic-based implementation, by talking with SEMM managers who supervised clinic delivery of SEMM, we identified the importance for clinic leaders to understand their patient population's needs and to prioritize SEMM delivery, focusing on current patients (in-reach recruitment strategy), or focus on delivering SEMM to women in surrounding communities to enroll women in the SEMM intervention (outreach recruitment strategy). Table 2 presents an example of findings from the rapid qualitative analysis of interviews conducted at the leadership level.

**Table 2:** Example findings of leadership barriers and facilitators from rapid qualitative analysis.

**Potential barriers**	**Potential facilitators**
Readiness construct(s) • Staff capacity (e.g., expanding CHWs role, hiring new CHWs) • Complexity- related to risk management and data protection (e.g., who will be responsible for upkeep of data and securing it)	Readiness construct(s) • Intra-organizational relationships (e.g., obtaining clinic staff buy-in)
	SCT construct(s) • Positive attitudes about CHWs being instrumental (e.g., CHWs are able to “wear multiple hats”) • Positive attitudes about having a program champion (e.g., program champion is critical for the success of the innovation)

### Task 2. Identify pre-implementation, implementation and maintenance outcomes, performance objectives, and determinants, and create matrices of change

Results of the needs and assets assessment helped inform the expected pre-implementation, implementation, and maintenance outcomes and to develop a list of specific actions, referred to here as performance objectives (POs), that each potential implementer (e.g., clinic leader, SEMM champions, CHW, and health coach navigator) needs to perform at each of the implementation stages (see Tables 3.1–3.3). Direct feedback from the clinic staff confirmed that implementation and maintenance of the SEMM intervention as a standard practice would require the endorsement of clinic leadership and commitment of resources, including an emphasis on dedicated personnel time.

**Table 3.1:** Implementation outcomes and performance objectives: leadership level (example).

**Implementer**	**Implementation outcome**	**Performance objectives**
Clinic leadership	Clinic leadership will support implementation of the SEMM intervention.	1. Review SEMM intervention objectives, components, experiences of other clinics, and identify relative advantages of implementing SEMM 2. Evaluate clinic needs: Note clinic BCS and CCS, and HPV vaccination rates 3. Communicate with and obtain buy-in from the Board/clinic leadership 4. Communicate the benefits of implementing SEMM to clinic staff. • PO4a. Talk informally to the staff about the importance of SEMM • PO4b. Use effective communication style (clear, coherent, and consistent communication) to support SEMM implementation • PO4c. Use data on SEMM effectiveness to persuade clinic staff of program importance • PO5d. Inform staff about how SEMM will help improve performance on their BCS and CCS quality measures 5. Communicate to the clinic staff that implementing SEMM is a priority 6. Determine clinic's high-level goals and goals for implementing SEMM (i.e., # of women recruited, # educated, # navigated, and # screened) 7. Identify resources (e.g., budget, space for education sessions, and staff time to complete training and implement SEMM) 8. Build relationships with key external stakeholders to support community outreach (e.g., local CBOs that serve the target population, state/county Public health officers, etc.) 9. Receive and report program updates to Board to ensure alignment to clinic goals
	Clinic leadership will maintain delivery of the SEMM intervention in their clinic	1. Discuss and seek funding approval 2. Identify opportunities for technical assistance and additional staff training

**Table 3.2:** Implementation outcomes and performance objectives: program manager/champion level (example).

**Implementer**	**Implementation outcome**	**Performance objectives**
SEMM program manager and/or champion	SEMM program managers and/or champions will support and motivate CHWs to deliver the program	1. Train CHWs to deliver SEMM 2. Communicate to CHWs that by implementing SEMM they are helping women in their community increase prevention and early detection of cervical cancer and early detection of breast cancer 3. Facilitate regular CHW meetings to debrief CHWs, coordinate implementation, and identify areas of need for retraining to build CHW capacity 4. Communicate summary reports to CHWs regarding numbers of women reached and served by SEMM (e.g., numbers of women screened or completion of HPV vaccinations as a result of CHW work)

**Table 3.3:** Implementation outcomes and performance objectives: *Promotora*/CHW/Health Coach Navigator level (example).

**Implementer**	**Implementation outcome**	**Performance objectives**
CHW/*Promotora*/Health Coach Navigator	*Promotora/*CHW/Health Coach Navigator will support implementation of *Salud en Mis Manos* (*SEMM*) to improve breast and cervical cancer screening (BCS, CCS) and HPV vaccination among eligible Latinas (21–65 years)	PO1. Understands the goals, purpose, objectives, and target group of SEMM intervention • PO1a. Participates in SEMM training • PO1b. States importance of screening and vaccination for early detection and control of cervical and breast cancer • PO1c. Learns how to use the SEMM data tracking system to record data • PO2. Understands the importance of her role as CHW in the SEMM intervention • PO2a. Identifies eligible Latinas through in-clinic and outreach • PO2b. Screens, enrolls eligible and interested Latinas and takes informed consent • PO2c. Collaborates with external stakeholders and partners for outreach PO3. Delivers education sessions with fidelity using SEMM intervention materials PO4. Assesses participants' readiness, intention, and barriers to get screened or vaccinated (Health Coach Navigation)

Insights of research team members with previous and current experience managing implementation of the SEMM intervention were leveraged to help identify implementer-specific POs. For example, the POs of a designated manager related to providing guidance and support to CHWs, such as developing CHWs' clinic-based recruitment or community-based outreach plans. Other manager POs related to facilitating routine CHW meetings to address challenges and share successes, to provide continuous process monitoring to ensure CHWs reach under-screened or unvaccinated women most in need of the SEMM education and navigation support, and to sustain CHW motivation for the work.

Finally, the research team reviewed each implementer's POs and finalized the list of POs for clinic leaders, SEMM program managers and/or champions, and CHWs. Review of the POs by current intervention implementers led to the identification of missing and overlapping tasks. Tables 3.1–3.3 present examples of SEMM implementation and maintenance POs describing the specific actions for implementers (clinic leadership, SEMM program manager and/or champion, and CHWs). For the clinic leaders, for example, POs were identified by asking “*What does the clinic leadership need to do to garner clinic Board of Directors' commitment of resources to support the program? What do clinic leaders need to do to plan the staffing to manage and deliver SEMM*?”

Next, the research team identified factors influencing implementation and developed the matrices of change objectives by crossing the selected behavioral and organizational determinants with identified performance objectives asking the question, “*What needs to change for the implementers to accomplish the specific implementation performance objective*?” The research team also considered behavioral science theories (e.g., SCT) and implementation science frameworks (e.g., ISF) in the identification of determinants and development of matrices of change (Table 4). For example, the ISF domain, “motivation,” guided the selection of specific attitudinal determinants expected to influence implementation and maintenance of the program. These included subconstructs, such as relative advantage, potential fit or compatibility, and the SEMM intervention's effectiveness in improving an important health problem prioritized by the clinic leadership (e.g., low cervical cancer screening rates and HPV vaccination rates). All ISF, and Readiness constructs from the R = MC^2^ heuristic (readiness= motivation × innovation specific capacity × general capacity) informed the types of implementers that may need to be involved to support implementation and deliver the program as well as the types of capacity needed for implementation to be successful (15). These matrices of change objectives served as the roadmap for designing the SEMM-DIA implementation strategies. Table 4 presents an example matrix for clinic leadership.

**Table 4:** Matrices of change for implementation (example): clinic leadership.

**Performance objective**	**Determinant**					
	**Attitude**	**Knowledge**	**Skills and self-efficacy**	**Outcome expectations**	**Feedback and reinforcement**	**Normative beliefs**
Clinic director will review SEMM objectives, components, experiences of other clinics, and relative advantages of implementing SEMM	AT1a. Believe that SEMM fits with organizational priorities	K1a. Describe SEMM as an evidence-based intervention for Latinas that was shown to be effective in increasing BCS and CCS among Latinas (21–65 years)	SSE1a. Feels confident in identifying SEMM components to share with team members based on clinic role	OE1a. Expect that implementing SEMM will increase guideline recommended BCS, CCS, and HPV vaccination rates among Latinas	OB1a. Believe that by implementing SEMM clinic demand for services will increase	NB1a. Recognize that other clinics review program objectives, components, and relative advantage before implementing a new cancer prevention program
	AT1b. Review SEMM components, materials, experiences of other clinics implementing SEMM in a favorable manner	K1b. Recognize SEMM is culturally appropriate	SSE1b. Feels confident in using SEMM-DIA to identify SEMM materials to share with clinic staff	OE1b. Expect that by providing staff and patients with information SEMM uptake will be achieved	OB1b. Believe that by implementing SEMM Texas will achieve Healthy People 2030 goals	NB1b. Believe that the other clinic systems that implemented SEMM had successfully implemented it
	AT1c. Believe SEMM is better suited for the clinic compared to other programs and/or usual practice	K1c. Describe that SEMM is available at no cost		OE1c. Expect that patients will use SEMM information for BCS, CCS, and HPV vaccine uptake	OB1c. Expect that by knowing the experiences of other clinics that have implemented SEMM, s/he will be able to evaluate the pros and cons of adopting/implementing SEMM	
	AT1d. Believe that SEMM has unique components and benefits that make it relevant for the community	K1d. Recognize that the program will provide resources to the clinic and CHWs		OE1d. Believe that the SEMM intervention will improve BCS, CCS and HPV vaccination rates among participating women	OB1d. Expect that by knowing the experiences of other clinics that have implemented SEMM will help successfully implement SEMM	
	AT1e. Believe that SEMM meets the standards of previously implemented programs	K1e. Describe the program as a tool for increasing BCS, CCS, and HPV vaccination among Latinas (21–65 years)				
	AT1f. Recognize that other clinics have successfully implemented SEMM	K1f. Describe potential availability of CHWs to deliver SEMM				
	AT1g. Believe that SEMM is an easy program to implement and will serve the needs of the community	K1g. Describe the steps needed to adopt and implement SEMM				
	AT1h. Believe that SEMM is an easy program to implement in clinic settings	K1h. Describes patient education needs				
		K1i. Describe SEMM components and advantages				

### Task 3. Select theoretical methods and identify or design implementation strategies

The planning group selected evidence-based methods based on the targeted determinants and performance objectives, as well as informed by types of methods that have worked before to address identified implementation challenges (e.g., such as potential lack of motivation, capacity of staff to manage or deliver the program). For example, to address the potential skills and self-efficacy required of CHWs to implement SEMM, the team identified implementation strategies to target CHW training needs, targeting potential implementation threats (see Table 5). For example, the team identified the need to provide video testimonials of CHWs with previous experience implementing SEMM in their clinics. The research team would design the testimonial to show a CHW discussing how the SEMM training helped them to learn to deliver the intervention, and as a result, the implementer's satisfaction of seeing that their delivery of SEMM helped women they served to complete their breast and cervical cancer screenings, and HPV vaccinations. The previous implementers would also share their perspectives regarding the types of supporting materials and protocols (e.g., simple) that enabled CHWs to learn to deliver education and navigation support to patients. The testimonials also would include patients sharing their own positive experience with SEMM.

**Table 5:** SEMM-DIA program implementation intervention plan (example).

**Agent/ Implementer**	**Determinants**	**Implementation strategies**		
		**Theoretical change methods**	**Practical applications of methods**	**Component**
Clinic leadership and/or Program manager/champion	- Awareness/perceptions - Outcome expectations - Skills and self-efficacy - Feedback and reinforcement	- Persuasion - Modeling	- Informational video describing SEMM goals, components, and benefits - Video testimonials of clinic leaders discussing how/why they implemented SEMM in their clinics - Video/animated tutorial for implementers - Program manager/champion train-the-trainer guide	- SEMM-DIA program orientation session - Program tracking tools (online and/or electronic) - SEMM manager training - Technical assistance
		- Communication - Mobilization	- Technical assistance *via* tele-mentoring platform Project ECHO - E-newsletter template to engage with stakeholders	
	*Characteristics of the innovation*: - Relative advantage - Comparability - Complexity - Trialability	- Organizational consultation planning - Advanced organizers - Environmental reevaluation	- SEMM implementation inventory/implementation readiness checklist—for assessing clinic resources (personnel and infrastructure) - Roles and Responsibilities SOP manual: for SEMM manager/champion and CHWs - Program implementation guide, clinic handbook - Quality monitoring tools and systems	
CHW	- Awareness/perceptions - Outcome expectations - Skills and self-efficacy - Feedback and reinforcement	- Information - Persuasion	- Informational video on benefits of implementing SEMM - Video testimonials of CHWs discussing implementation benefits and challenges	- SEMM-DIA online tool - Program orientation session - CHW online training - Technical assistance
		- Technical assistance/capacity building - Facilitation	- Program implementation guide, Clinic handbook - SEMM implementation inventory/implementation readiness checklist—for assessing clinic resources (personnel and infrastructure) - CHW Training manual/curriculum - SEMM in-reach/outreach strategy toolkit - Technical assistance *via* tele-mentoring platform Project ECHO - Collaborator manual to support implementation	
		- Skill building - Guided practice - Vicarious reinforcement	- Computer assisted SEMM training scripts	

Based on their influence on determinants (e.g., attitudes, self-efficacy, and skills; see Table 4) and contextual factors, guided by SCT, the team identified behavioral change methods (e.g., modeling verbal persuasion, and communication). These methods were operationalized to guide adaptation of the existing CHW manager trainings. For example, CHW manager trainings included a train-the-trainer guide with step-by-step demonstrations of how to facilitate CHW peer learning (e.g., modeling). Trainings were adapted to build the CHW manager's capacity to supervise CHW delivery of SEMM, and to facilitate peer learning and peer support strategies during regular CHW team meetings. Empowerment and support of CHWs, managers and leadership were also addressed by planning testimonials based on positive experiences of previous program implementers who share benefits of promoting the intervention within their clinic systems (e.g., helping to meet performance measures for cervical cancer screening) and benefiting their communities by addressing high priority problems in vulnerable communities. The implementation support planning process, therefore, not only provided practical support (e.g., knowledge and resource transfer to potential users), but also included implementation strategies and theoretically informed methods to help address both implementation challenges and user-related determinants of implementation (e.g., capacity to deliver SEMM, outcome expectations that SEMM will help women they serve to complete screenings and HPV vaccinations, and motivation to implement the program).

### Task 4. Produce implementation protocols and materials to guide intervention implementation

The fourth task of the Implementation Mapping process included designing the SEMM-DIA implementation strategy materials, protocols, and training. This involved describing the SEMM-DIA design document, creating the SEMM-DIA resource inventory, designing the SEMM-DIA website, and programming the SEMM-DIA website.

#### SEMM-DIA design document

**Figure 2:**
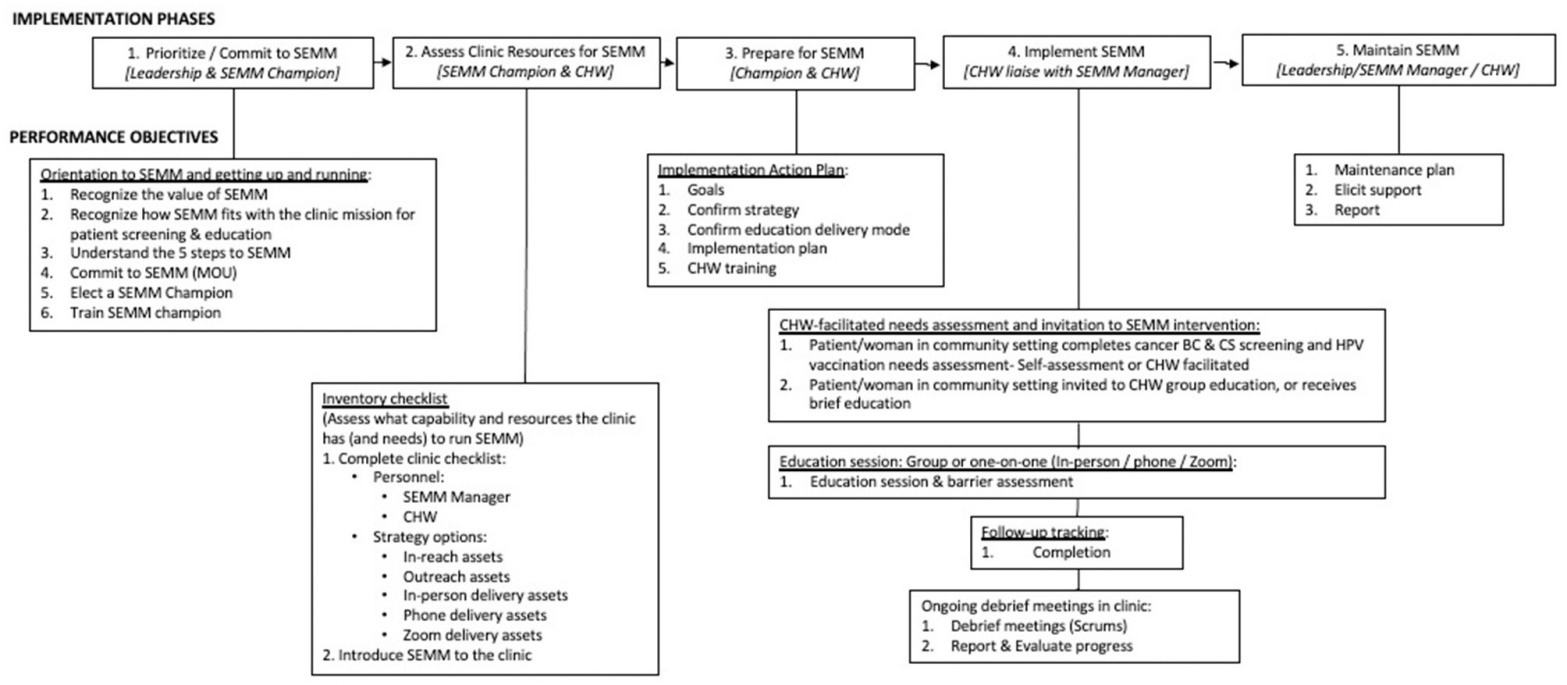
SEMM-DIA design document.

SEMM-DIA is a multi-faceted multi-component implementation strategy. The SEMM-DIA design document was derived from the matrices of change objectives developed in Task 2 (Figure 2). It represents a top-level conceptualization of how SEMM-DIA functions. The performance objectives were ordered in a chronological sequence according to when they would occur during implementation (Table 6). On review, these performance objectives suggested a natural clustering that corresponded approximately to the Exploration, Preparation, Implementation, and Sustainment (EPIS) framework (14). This overriding “meta” implementation framework comprised five phases to support SEMM implementation and maintenance. These phases were (1) Exploration (prioritizing SEMM), (2) Preparation (assessing clinic readiness), (3) Preparation for implementation of SEMM, (4) Implementation, and (5) Sustainment (or maintenance). Each phase was associated with clinic personnel responsible for that phase (i.e., clinic leadership, SEMM champion, CHWs and health coach navigators).

**Table 6:** SEMM-DIA resource inventory (example for phase 1, step 1).

**PHASE 1. Prioritize/commit to SEMM in your clinic**						
**I**	**II**	**III**	**IV**	**V**	**VI**	**VII**
**Step**	**Program phase tasks (performance objectives and change objectives)**	**Agent**	**Methods**	**Implementation strategies (methods and components)**	**SEMM implementation resources**	
					**Existing**	**Pending (to produce)**
1	PO1. Leadership reviews SEMM intervention objectives, components, experiences of other clinics, and recognizes the relative advantages of implementing SEMM Change objectives: AT1a-j, K1a-d, OE1a-d, OB1a-d	Leadership	M1. Environmental reevaluation M2. Framing M3. Cultural similarity M4. Modeling M5. Persuasive communication M6. Goal setting M7. Belief selection	1. Video introducing the SEMM intervention, components, and benefits (M:1–7; online tool) 2. Program implementation guide and clinic handbook (M:1–7; program orientation session)| 3. Clinic SEMM needs and resources assessment checklist (M:1–7; online tool) 4. Implementation readiness checklist/SEMM implementation inventory (M:1–7; online tool, program orientation session) 5. SEMM clinic example workflow (M:1–7; online tool, program orientation session)	1. Collaborator agreement form 2. SEMM recorded presentation 3. Overview materials/toolkits included in SEMM	1. Update SEMM-DIA presentation 2. Update SEMM-DIA MOU scope of work

The SEMM-DIA design document lists performance objectives embedded within this framework in thematic clusters representing: (1) Orientation; (2) Inventory checklist (for the implementer to assess delivery capacity and patient/community outreach needs); (3) Clinic Implementation Action Plan; (4) SEMM components: CHW-delivered education and referrals and health coach navigator-delivered barrier mitigation to help women overcome personal and system-level barriers to accessing and using clinic services; and (5) Maintenance planning (Figure 2). This provides a context for when the performance objective occurs within the SEMM-DIA implementation process. Each performance objective refers to resources that are required to complete the objective, represented as row numbers within the SEMM-DIA resource inventory.

#### SEMM-DIA resource inventory

**Figure 3:**
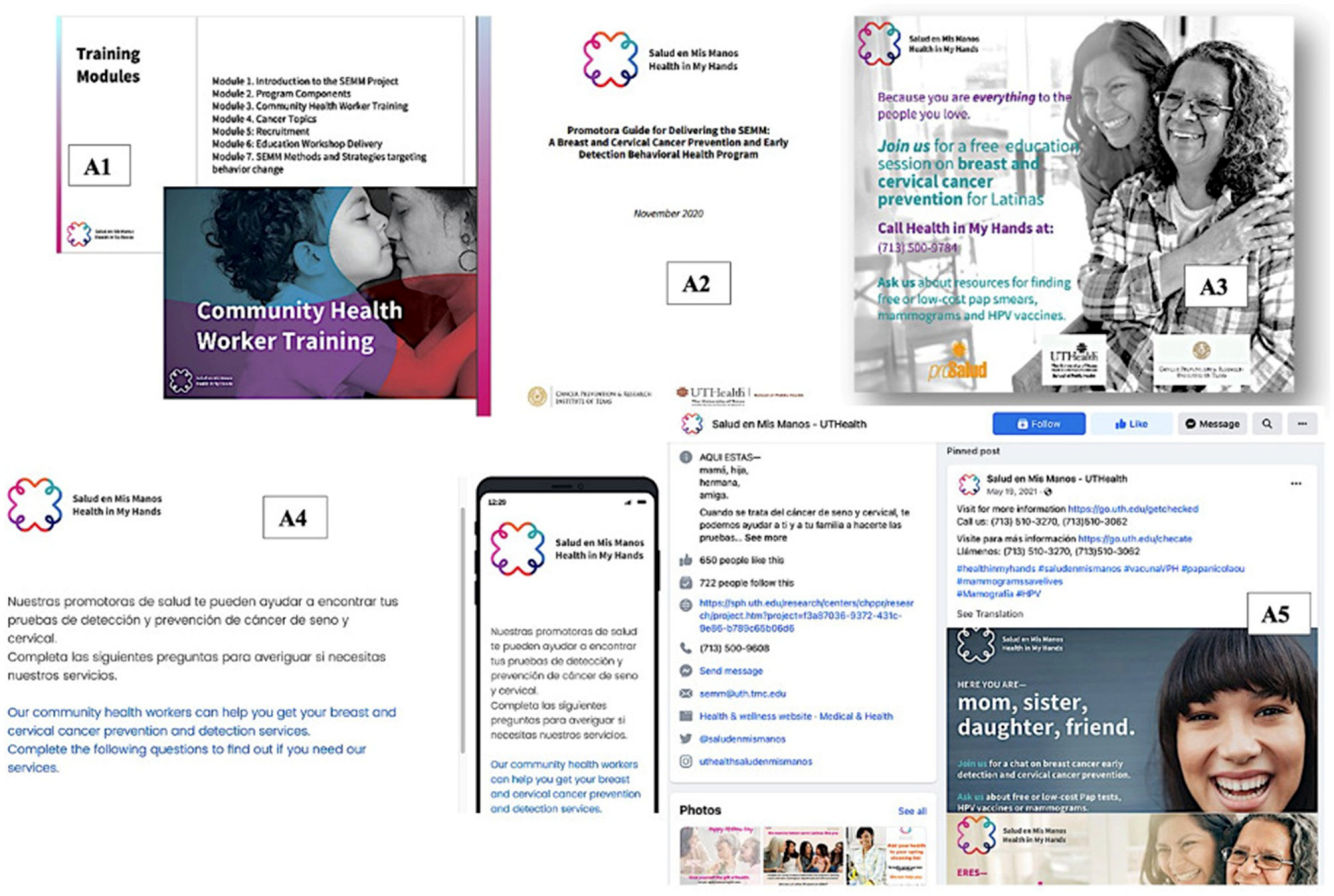
Sample of existing SEMM Resources to Support CHW Training, Community Outreach, and Recruitment to the SEMM Intervention: **(A1)** CHW Training Curriculum, **(A2)** CHW Guide for Delivering SEMM Education Intervention, **(A3)** SEMM Promotional Print Material, **(A4)** SEMM Participant Needs Assessment, and **(A5)** SEMM Social Media.

The SEMM-DIA resource inventory lists the resources that enable clinic personnel to complete each performance objective in SEMM-DIA (Table 6). The inventory provides information on the phase and performance objectives, agent (responsible clinic personnel), methods and strategies (from Step 3), and the SEMM implementation resources. The resources include written information about SEMM, a clinic inventory form to assess readiness for SEMM, a training curriculum for SEMM champions and CHWs, a template SEMM preparation plan, a CHW screening and tracking form, CHW patient and community awareness educational materials, and template maintenance plan (Figure 3). The resources are categorized as either “Existing” implementation resources (those implementation materials that had already been developed) that could be adopted or adapted, (such as CHW delivery guides) or as “Pending” resources (those in need of development; Table 6, Columns 6 and 7). This provides guidance on what pre-existing SEMM resources (again see Figure 3) could be leveraged in the SEMM-DIA development effort and to identify the extent of resource development required.

SEMM-DIA implementation support resources were designed to align with the varied implementation delivery goals, including if the priority for SEMM implementation was on “In-reach” (engaging existing patients within a clinic), or “out-reach” (engaging the broader community). Further materials, and tools were designed to facilitate varying delivery modalities including CHW-mediated one-on-one or group-based SEMM education and varying delivery channels including in-person, phone-based, or video-conference platforms.

#### SEMM-DIA website design

The SEMM-DIA website was designed to be a multi-faceted multi-component implementation support strategy to guide planning and implementation of the SEMM EBI. A design document was developed to be the “blueprint” to guide construction of the SEMM-DIA website. The document was informed by the previous implementation planning tasks and describes the website's purpose and context, functional parameters (protocols, activities, and flow), design features, and resources (associated materials and assets to support adoption, implementation, and maintenance).

**Figure 4:**
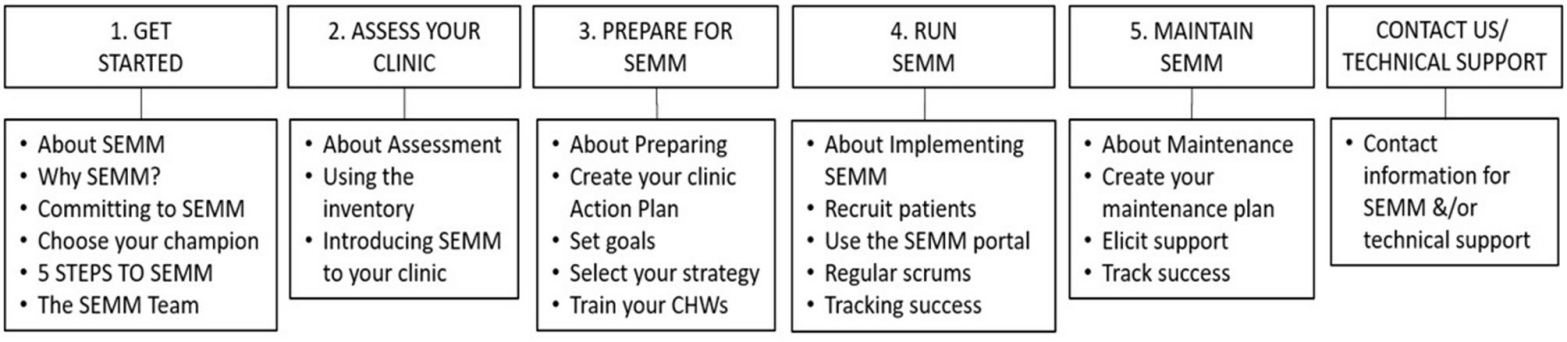
SEMM-DIA website structure.

The SEMM-DIA website was designed as an asynchronous, easily accessible, and user-friendly online guide and reference to SEMM implementation. The website guidance was designed to support navigation through the “5 steps to SEMM” in accordance with the SEMM-DIA design document (Figure 4). Development was also informed by clinic staff's preference for a simple, form-based approach that could be easily integrated into CHW workflow. They preferred to be able to download needed forms for use in the clinic or community rather than use of mHealth or technology-dependent applications for real-time use with patients (e.g., electronic data collection surveys or decision support tools). Thus, to enable accomplishment of each step the website was designed to provide SEMM resources for download (e.g., pdf forms that are the core of the SEMM screening and education for CHWs to use as hardcopy versions) or streaming (e.g., testimonial videos) in a manner that provided context and rationale for use within the SEMM-DIA design document. The website was designed to accommodate the needs of relevant clinic stakeholders including clinic leadership, SEMM champions, and CHWs.

#### SEMM-DIA website programming

SEMM-DIA website programming was guided by the design document which provided the specifications previously described, priority audience (e.g., program adopters, SEMM champions, CHWs, or health coach navigators), scripts (e.g., for video testimonials planned for creation), and images (e.g., stock photos, or existing program photographs). In addition, a SEMM-DIA description, and specific instructions of each element in the SEMM-DIA plan, were provided to the SEMM-DIA website developers. This included the existing graphic design assets to retain the same look and feel of the original intervention design. Figure 3 provides a sample of the SEMM material design “look and feel,” as used in existing SEMM CHW training curriculum, recruitment, and community outreach materials.

The Implementation Mapping planning process helped incorporate guidance from theoretical frameworks and informed the design and content of all the SEMM-DIA implementation strategies, including the SEMM-DIA website, as well as technical assistance strategies such as an initial program orientation session with clinics (either in-person or virtually), the SEMM-DIA Project ECHO tele-mentoring series, and IMAdapt.org to support EBI and implementation strategy adaptation. These additional individual technical assistance strategies are accessible *via* the online SEMM-DIA website. The implementation strategies embedded within the SEMM-DIA website component are the ones highlighted in this paper.

### Task 5. Evaluation the implementation outcomes

In Task 5, design of the evaluation plan focused on determining the effect of the SEMM-DIA implementation strategy on implementation outcomes, as well as the overall effectiveness of the SEMM intervention on increasing breast and cervical cancer screening and HPV vaccination rates. We will conduct a hybrid type 2 effectiveness-implementation group randomized trial to determine effectiveness and compare the effect of SEMM-DIA vs. Usual Implementation Practice (usual practice) on Reach, Effectiveness, Implementation, and Maintenance of SEMM, focusing on intentions to maintain SEMM due to the time constraints on evaluating long-term maintenance outcomes (25). These primary outcomes (four of the five RE-AIM dimensions) are defined in Table 7. A cost-effectiveness analysis to produce data on the economic details of SEMM-DIA implementation in clinic practice settings is planned as part of a future phase of this study.

**Table 7:** RE-AIM framework utilized constructs defined.

**Construct**	**Definition**
Reach	Proportion of women who participate in the SEMM education session among those eligible
Effectiveness	(For screening and HPV vaccination) % of women who complete screening or vaccination among all eligible women participating in the program
Implementation	Extent to which SEMM program components were used
Level of implementation	Number of implementation steps that have been carried out
Implementation fidelity	Degree to which SEMM components are implemented by CHWs as prescribed (degree of implementation for each CHW; frequencies and proportion of CHWs performing the required behaviors; proportion of patients recommended for screening and HPV vaccination)
Implementation dose	Time spent in education sessions; # of navigation calls
Maintenance intention	Intention to implement the program in the next 6 months

To guide the overall evaluation, the planners articulated implementation evaluation questions to assess whether the SEMM-DIA implementation strategy influenced implementation determinants and outcomes such as fidelity of the SEMM implementation plan. Other implementation questions included whether SEMM-DIA was acceptable to the program implementers (e.g., implementer satisfaction), and did SEMM reach the priority implementers as planned. At the SEMM intervention level, process evaluation questions focused on whether each implementer delivered the intervention as planned (e.g., assessing fidelity of CHW implementation of SEMM), and whether the intervention reached the intended priority population (e.g., women overdue for breast and cervical cancer screenings, or HPV vaccination).

Implementation facilitators and barriers identified in Task 1 (needs and assets assessments) helped to identify potential mediators and moderators for the evaluation plan. In the selection of determinants and the development of matrices of change objectives, which focused on “*what needs to change in the determinants (e.g., attitudes, skills, knowledge)*,” the research team had considered behavioral science theories, such as SCT. Consequently, the evaluation plan also selected measures to evaluate targeted individual-level constructs identified, such as to evaluate the effect of CHW training on implementers' knowledge, skills, self-efficacy, and outcome expectations.

In addition, implementation science frameworks (e.g., Integrated Systems Framework; ISF) that guided synthesis of the formative work conducted in Task 1, and informed implementation planning, consequently informed evaluation planning. Specifically, we identified the contextual factors of implementation to include in the evaluation plan at the organizational level, such as organizational readiness. Using the heuristic for organizational readiness—motivation × innovation-specific capacity × general capacity, (R = MC^2^) from ISF—we identified important constructs to include in the evaluation plan related to motivation (e.g., relative advantage, compatibility, complexity, priority), general capacity (e.g., culture, resource utilization, leadership, staff capacity), and innovation-specific capacity (e.g., knowledge/skills/abilities, program champion). Thus, the matrices developed in tasks 1–4 served as a road map to guide development of the evaluation plan.

**Figure 5:**
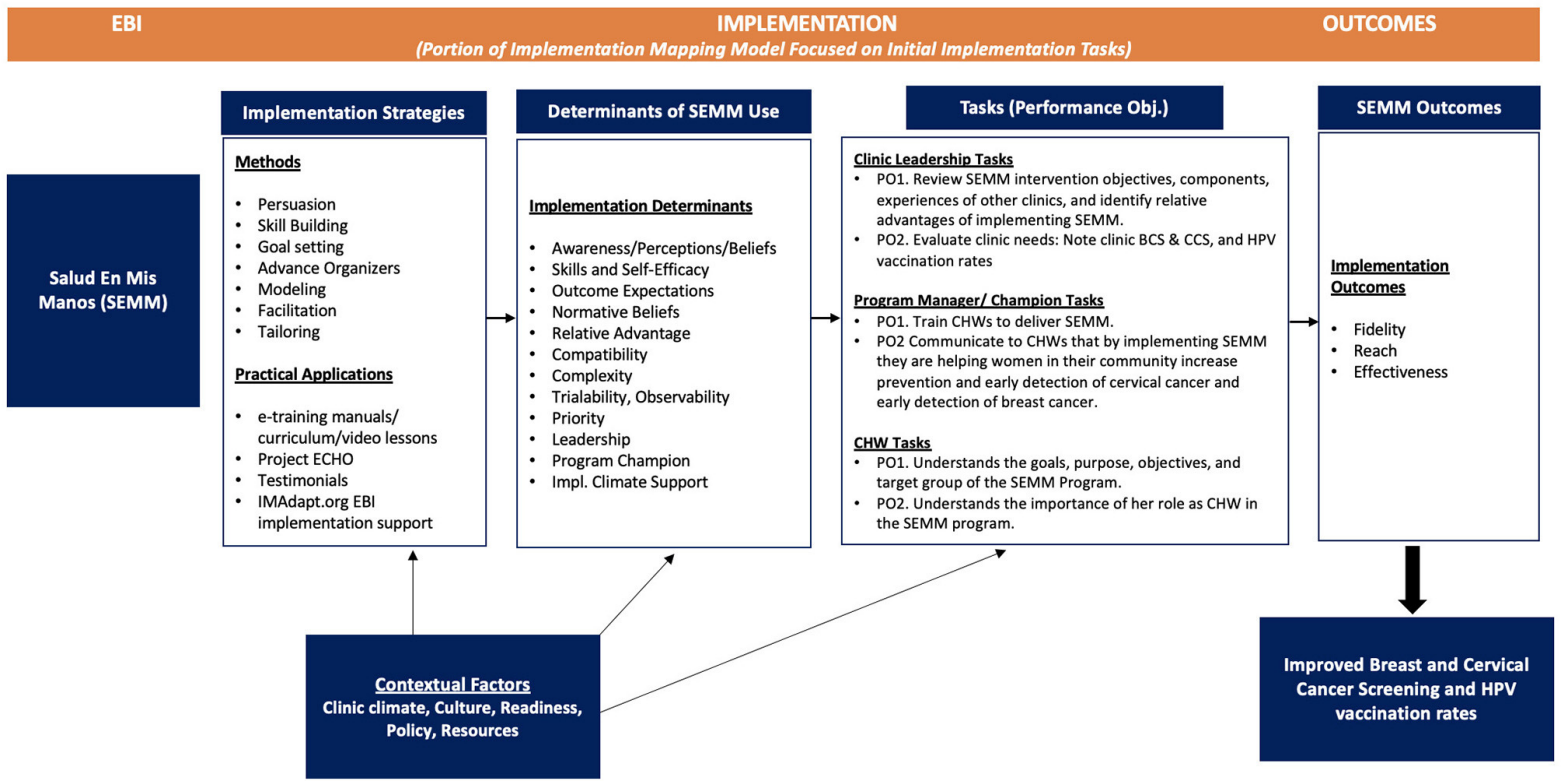
SEMM-DIA Implementation Mapping logic model.

Finally, the research team developed a logic model to provide a graphical representation of how strategies influence implementation and effectiveness of outcomes as part of the process for planning implementation strategies (Figure 5). This Implementation Mapping logic model illustrates the planned linkages between the implementation strategy, mechanisms, determinants of implementation, and proximal and distal implementation outcomes, thus helping describe the SEMM-DIA strategy's mechanisms of change. The logic model begins with the intervention (SEMM) on the far left and progresses to implementation strategies that deliver change methods. The research team designed these strategies to influence determinants, which in turn effect change in the implementation behaviors and conditions, leading to implementation.

This logic model thus helps to define our SEMM-DIA implementation outcomes as well as SEMM effectiveness outcomes (breast, cervical and HPV vaccination), to be examined in the planned hybrid type 2 study. The comprehensive SEMM-DIA implementation support plan facilitates implementation of the SEMM intervention as planned (increasing implementation fidelity) among a priority population in need of the program (increasing efficiency in reach, minimizing over-inclusion and under-inclusion of the target population). The logic model also represents how the plan results in an intervention that effectively increases breast and cervical cancer screening, and HPV vaccination among underserved Latinas. Finally, the logic model is especially useful for communicating both the evaluation outcomes, and the causal mechanisms of the SEMM implementation and evaluation plan to non-academic clinic or community partners.

## Discussion

Effective and feasible implementation strategies are needed to increase the use of evidence-based cancer prevention and control interventions in community and health care settings. Implementation support is also needed to promote implementation with fidelity to retain effectiveness when EBIs are translated from research to practice. This paper described the use of Implementation Mapping to plan a multifaceted implementation strategy for the delivery of an effective breast and cervical cancer screening intervention targeting Latinas. The development of SEMM-DIA provides an opportunity to illustrate how Implementation Mapping can help implementation strategy planners use theory, evidence, and community engagement to inform strategy selection and tailoring. The use of Implementation Mapping also results in a logic model that presents a graphic depiction of the planned linkages between the implementation strategy, mechanisms, determinants of implementation and proximal and distal implementation outcomes, helping to describe the SEMM-DIA strategy's mechanisms of change.

A major strength of this work is that it provides a model for developing a multi-component, multi-level implementation support strategy to enable the implementation of a CHW delivered intervention in clinical settings (26, 27). CHW-delivered peer-to-peer behavioral interventions and patient navigation are recognized strategies to increase access to and utilization of preventive health care services, serving as effective approaches to increase parity for medically underserved ethnic and racial minorities (28–37). However, there are notable gaps in implementation and maintenance of such EBIs. The Implementation Mapping process used to plan SEMM-DIA provides a model to help identify common challenges to implementation and maintenance specific to CHW-delivered interventions, and provides an example for strategies selected, or designed, to address these CHW intervention-specific implementation challenges. Strategies identified may benefit other CHW-delivered interventions, these include: (1) provide online CHW training materials to help maintain continuity of the program when there is CHW turnover, (2) embed materials developed to promote the program to reduce difficulty accessing materials in a timely manner, and (3) provide CHW manager training materials, to develop manager capacity to deliver CHW training, and provide continuous support and motivation to CHWs (9, 28, 38).

Another major strength of this work is the integration of multiple stakeholders in the planning process, using a collaborative approach (39–43). Implementation Mapping includes, as a foundational principle, the integration of implementers, community partners and other interested parties in the strategy development process. This includes people with experience delivering and managing the SEMM intervention (or similar CHW interventions), as well as other stakeholders and implementers (e.g., clinic leaders at FQHCs, clinic managers, and CHWs working in clinic settings). The importance of integrating stakeholders with extensive experience delivering and managing SEMM-specifically also helped to identify potential problems future implementers might encounter, and thus helped develop and select needed implementation support strategies. The SEMM-DIA planning team members with extensive experience managing and delivering SEMM provided their perspectives to planning and design decisions, such as identifying existing protocols and materials and resources that proved successful in supporting implementation that were leveraged in the design of SEMM-DIA. By engaging stakeholders with different roles and from different clinic settings, we were able to develop relevant and feasible methods and strategies with consideration of multiple perspectives and contingencies, ensuring that the implementation strategies addressed the needs and resources of the different organizations and the communities they served. Thus, throughout the process, we provided tailored options within the implementation strategies to influence different determinants and performance objectives for different types of users. The approach helped maximize generalizability of the SEMM-DIA design to a variety of potential users, as well as to diverse clinic and environmental contexts.

A challenge to this collaborative approach was scheduling meetings with clinic leadership and health care providers who often have competing priorities (e.g., during this study, clinic stakeholders' time was limited due to the COVID-19 pandemic). We learned that conducting regular virtual meetings with CAB members was essential to ensure inclusion of community stakeholders' perspectives. In addition, we used an iterative engagement process, circling back to different stakeholders to integrate their insights and feedback at key decision points, enabling participation by clinic coordinators and CHWs during the intervention development process, but also cognizant of their limited time.

The existing SEMM-DIA strategy is primarily focused on implementation and maintenance. Since this project works with clinics who have already expressed some interest in SEMM, the strategy does not include a major focus on clinic leaders' initial decision to adopt SEMM. The research team focused on designing SEMM-DIA to support the pre-implementation phase following initial adoption, to ensure SEMM alignment with the clinic organization's goals and capacity, as well as to facilitate SEMM implementation within their clinic organization. This assumed that the clinic leadership had made a decision to adopt SEMM. Thus, we focused on developing implementation support for clinic leaders, managers and CHWs rather than on supporting leadership in a decision process to adopt SEMM. Future research is needed to examine the effect of the SEMM-DIA intervention on promoting adoption of SEMM as well as program maintenance. Further, because the implementation support system is designed as a multi-component multi-faceted implementation strategy, primarily within an online website, CHWs with limited technology skills may have difficulty accessing it, increasing reliance on SEMM CHW champions to provide SEMM-DIA resources to CHWs. Planned pilot testing of SEMM-DIA will help identify initial challenges, and pilot results will be used to identify solutions and further refine the implementation support strategies. Finally, ongoing evaluation will examine SEMM-DIA implementation outcomes, such as usability, feasibility, and acceptability, and SEMM intervention outcomes (e.g., completion of overdue breast and cervical cancer screenings, and HPV vaccination). To understand the degree of implementation, and degree of engagement with the SEMM-DIA dissemination and implementation support strategy, we will continuously monitor program implementation and stakeholder (clinic implementers) engagement. We will assess use of implementation materials and resources by clinic implementers through surveys and in-depth interviews. For the intervention group (SEMM-DIA study arm), we will also analyze implementers and decision makers' user data captured by SEMM-DIA (e.g., use, pathways, and Google Analytics), to examine the level of engagement with this implementation assistance. Because each clinic may use the SEMM-DIA implementation support differently (selecting elements that they decide will help their organization to implement SEMM effectively in their own clinic context and for the population they serve), there is not a predetermined “right” way to use SEMM-DIA. Therefore, in this study we will seek to identify potential mechanisms by which SEMM-DIA promotes fidelity in implementation outcomes and effectiveness of SEMM. Implementation monitoring and evaluation of the use of the implementation strategies will inform future adaptations of SEMM-DIA. Future SEMM-DIA implementation research will also include an implementation planning goal to develop and evaluate implementation strategies focused on supporting SEMM adoption, and will monitor maintenance over a longer period, to further improve widespread diffusion of SEMM.

In summary, we used Implementation Mapping to plan SEMM-DIA, a multifaceted implementation strategy (set of strategies). This paper describes the application of Implementation Mapping to develop implementation support strategies embedded in the SEMM-DIA website to serve as an example of how a systematic protocol can help apply theory and evidence for implementation strategy selection and development, describe the expected mechanisms of action of implementation strategies, and provide a framework for evaluation of implementation and effectiveness outcomes. Importantly, this approach integrates theory, empirical evidence, and EBI stakeholders' perspectives to develop relevant methods and implementation strategies, as well as to promote fidelity of implementation in the new adoption context. To promote implementation of evidence-based behavioral interventions into community practice, increased reporting of processes used to select and tailor and develop implementation strategies are needed. This paper begins to fill that gap (44).

## Data availability statement

The raw data supporting the conclusions of this article will be made available by the authors, without undue reservation.

## Author contributions

MF, LS, and RS contributed to the conception and design of the study. LS, MF, PL, EA, CC, AA, and RS wrote sections of the first draft of the manuscript. LS, RC, and CC conducted interviews. CC and MA performed the qualitative analysis for the study's needs assessment. LS, EA, and MG summarized findings representing previous experience of SEMM implementers on the team. LS led final writing of the manuscript with substantive contributions from MF, RS, and CM. AA led final revision changes and responses to reviewers during the resubmission process and finalized all tables and figures. PL finalized all citations. LS and MF read and approved the final manuscript.
